# Complete mitochondrial genome of Korean catfish, *Liobagrus somjinensis* (Actinopterygii, Siluriformes, Amblycipitidae), from South Korea

**DOI:** 10.1080/23802359.2020.1715870

**Published:** 2020-01-24

**Authors:** Philjae Kim, Jeong-Ho Han, Seung Lak An

**Affiliations:** a Research Division, National Science Museum, Daejeon, Korea;; b Water Environmental Management Department, Korea water Resources Corporation, Daejeon, Korea

**Keywords:** Korean catfish, mitochondrial complete genome, *Liobagrus somjinensis*, phylogenetic analysis

## Abstract

The Korean catfish, *Liobagrus somjinensis*, was recorded in 2010 as a new species of genus *Liobagrus*. The complete mitochondrial DNA sequence of *L*. *somjinensis* was sequenced by next-generation sequencing (NGS) analysis. The assembled mitogenome was 16,526 bp in length and encoded 13 protein-coding genes (PCGs), 22 tRNAs, and 2 rRNAs. The gene arrangement, content, and total size were clearly identical with the congeneric species, *L*. *mediadiposalis*. Phylogenetic analysis based on nucleotide dataset, consisting PCGs and rRNA genes revealed the taxonomical relationship in species level among the genus *Liobagrus*.

The family Amblycipitidae belonging to the order Siluriformes is distributed in Southern and Eastern Asia (Chen and Lundberg 1995; Ng and Kottelat 2000; Kim and Park 2002; Wright and Ng 2008; Nelson et al. 2016). In Korea, among the family Amblycipitidae, only three *Liobagrus* species have recorded before. However, genus *Liobagrus* consisted of five species, *L. somjinensis* and *L. hyeongsanensis* were reported as new species in 2010 and 2015, respectively. The complete mitogenomes were revealed in three species: *L. mediadiposalis*, *L. andersoni*, and *L. obesus* (Kartavtsev et al. 2007; Lee et al. 2016; Park et al. 2017). Herein, we provided the complete mitogenome of *L. somjinensis* (MN756661) for examining the phylogenetic relationship of Korean *Liobagrus*.

In this study, a specimen of *L. somjinensis* (NSMK-FI00004) used in this study was collected from the investigation site (35°01′20.04″N, 127°20′43.03″E) on 19 August 2019, and deposited in the Natural History Laboratory of National Science Museum (Daejeon, Korea). Mitochondrial DNA was isolated from caudal fin using the Qproteome Mitochondria Isolation Kit (QIAGEN, Hilden, Germany) and DNeasy Blood & Tissue DNA isolation kit (QIAGEN). The mitochondrial DNA (NSMK-DN00004) was stored in a deep freezer (–80 °C) of the National Science Museum, Korea, until used. PCR product for NGS analysis was amplified using mitochondrial DNA by REPLI-g Mitochondrial DNA kit (QIAGEN). The amplified mitochondrial DNA was prepared to sequencing library using QIAseq FX single-cell DNA library kit (QIAGEN), and DNA library was sequenced by Illumina Hi-Seq 2500 platform (San Diego, CA, USA) in GnC Bio Co. (Daejeon, South Korea). The sequence assembly and gene arrangement were performed by Geneious Prime 2019.2.1 (Biomatters, Auckland, New Zealand).

The mitochondrial complete genome sequences of *L. somjinensis* (MN756661) was 16,526 bp in length and contained 13 PCGs, 22 tRNA genes, and 2rRNA genes. The gene arrangement was exactly the same as another species of Amblycipitidae. The overall nucleotide compositions were 30.2% A, 25.3% T, 28.6% C, and 15.9% G. All PCGs had initiation codons ‘ATG’, except for COI started with ‘GTG’. The six PCGs (ND2, COX2, COX3, ND3, ND4, CytB) had incomplete terminal codon ‘T’, and terminal codon of the five PCGs (COX1, ATP8, ATP6, ND4L, ND5) was ‘TAA’. Only two PCGs (ND1, ND6) had ‘TAG’ as the terminal codon.

Maximum likelihood tree with 1000 replicates was constructed using PhyML 3.1 with GTR + I + G model (Guindon and Gascuel 2003; Guindon et al. 2010; Darriba et al. 2012). The dataset for analysis comprised 13 PCGs of the total of 35 Siluriformes mitochondrial genome; and four Gobiiformes mitochondrial genomes for outgroup. According to the ML tree, *L. somjinensis* was clearly distinct from *Liobagrus* species, and each of the families belonging to order Siluriformes formed a monophyletic clade. The phylogenetic tree indicated that molecular result was consistent with morphological classification (Figure 1).

**Figure 1.**
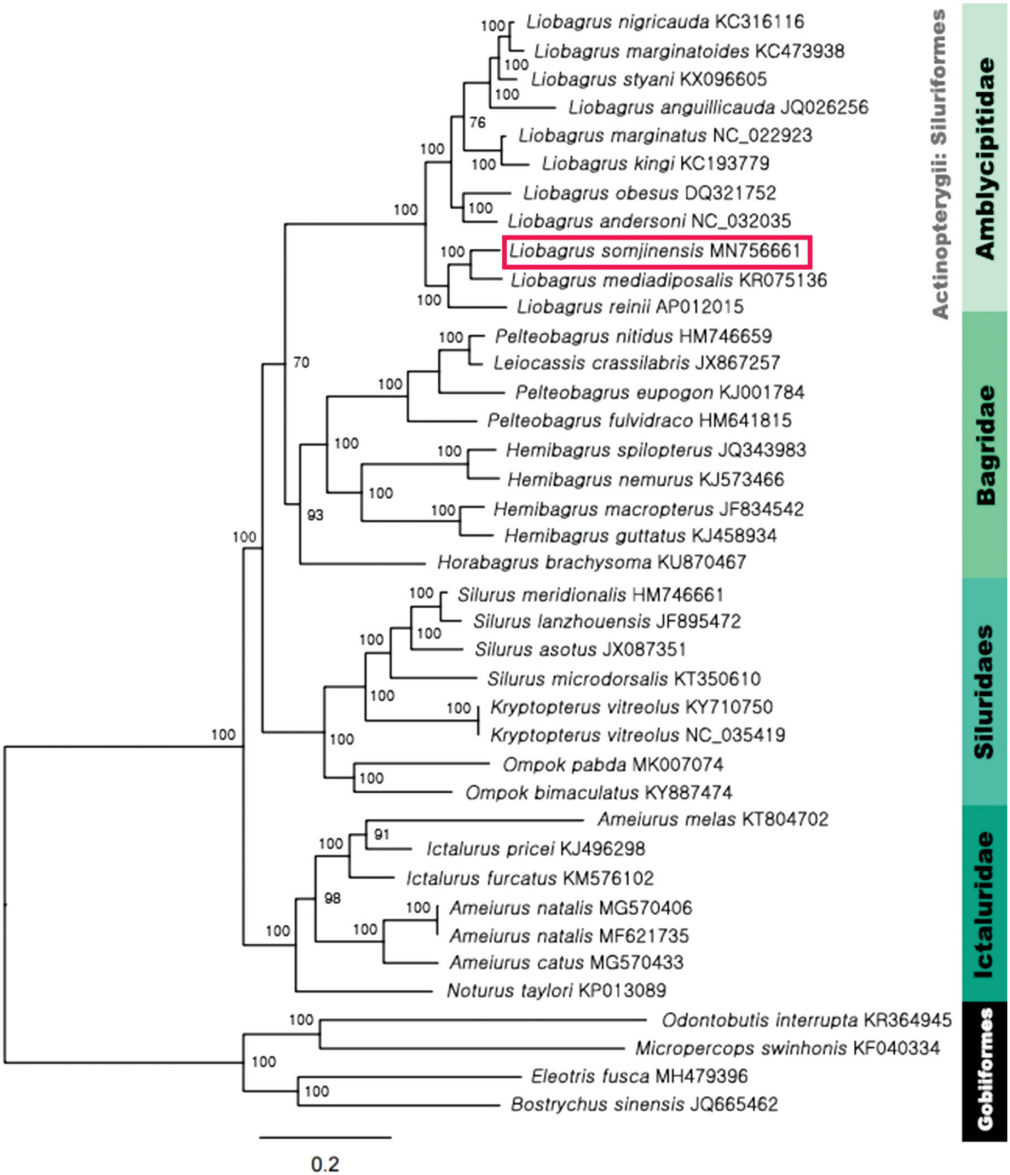
The phylogenetic position of *Liobagrus somjinensis*. Phylogenetic tree was constructed by maximum likelihood method and GTR + I + G based on the nucleotide sequences of 13 PCGs and 2 rRNAs of 33 Siluriformes species, included *L. somjinensis* (MN756661). Bootstrap support values are indicated on each node as >70.
